# Schizophrenia plausible protective effect of microRNA-137 is potentially related to estrogen and prolactin in female patients

**DOI:** 10.3389/fpsyt.2023.1187111

**Published:** 2023-08-23

**Authors:** Qian Peng, Zhun Dai, Jingwen Yin, Dong Lv, Xudong Luo, Susu Xiong, Zhijiang Yang, Guangmin Chen, Yaxue Wei, Ying Wang, Dandan Zhang, Lulu Wang, Debo Yu, Yusheng Zhao, Dele Lin, Zhiyu Liao, Yongxi Zhong, Zhixiong Lin, Juda Lin

**Affiliations:** Department of Psychiatry, Affiliated Hospital of Guangdong Medical University, Zhanjiang, China

**Keywords:** schizophrenia, estrogen, miR-137, single nucleotide polymorphisms, genetic association, gene expression *in vitro*, prolactin

## Abstract

**Background:**

Schizophrenia (SCZ) is a serious chronic mental disorder. Our previous case–control genetic association study has shown that microRNA-137 (miR-137) may only protect females against SCZ. Since estrogen, an important female sex hormone, exerts neuroprotective effects, the relationship between estrogen and miR-137 in the pathophysiology of SCZ was further studied in this study.

**Methods:**

Genotyping of single-nucleotide polymorphism rs1625579 of miR-137 gene in 1,004 SCZ patients and 896 healthy controls was conducted using the iMLDR assay. The effect of estradiol (E2) on the miR-137 expression was evaluated on the human mammary adenocarcinoma cell line (MCF-7) and the mouse hippocampal neuron cell line (HT22). The relationships between serum E2, prolactin (PRL), and peripheral blood miR-137 were investigated in 41 SCZ patients and 43 healthy controls. The miR-137 and other reference miRNAs were detected by real-time fluorescent quantitative reverse transcription-PCR.

**Results:**

Based on the well-known SNP rs1625579, the distributions of protective genotypes and alleles of the miR-137 gene were not different between patients and healthy controls but were marginally significantly lower in female patients. E2 upregulated the expression of miR-137 to 2.83 and 1.81 times in MCF-7 and HT22 cells, respectively. Both serum E2 and blood miR-137 were significantly decreased or downregulated in SCZ patients, but they lacked expected positive correlations with each other in both patients and controls. When stratified by sex, blood miR-137 was negatively correlated with serum E2 in female patients. On the other hand, serum PRL was significantly increased in SCZ patients, and the female patients had the highest serum PRL level and a negative correlation between serum PRL and blood miR-137.

**Conclusion:**

The plausible SCZ-protective effect of miR-137 may be female specific, of which the underlying mechanism may be that E2 upregulates the expression of miR-137. This protective mechanism may also be abrogated by elevated PRL in female patients. These preliminary findings suggest a new genetic/environmental interaction mechanism for E2/miR-137 to protect normal females against SCZ and a novel E2/PRL/miR-137-related pathophysiology of female SCZ, implying some new antipsychotic ways for female patients in future.

## Introduction

Schizophrenia (SCZ), a severe mental disorder with a lifetime prevalence of ~0.7% in populations worldwide, remains an etiological and therapeutic challenge (1). In terms of epidemiology, SCZ is modestly more common in men than in women, and its peak age of women is usually 3–4 years later than that of men, and there is a second peak age after menopause in women (1, 2). For these reasons, many scientists believe that the female sex hormone estrogen has a protective effect on SCZ by buffering females against the occurrence and severity of the illness (3, 4). Indeed, estrogen replacement therapy (ERT) has an adjunctive antipsychotic effect (5, 6). However, ERT is suboptimal in efficacy and has considerable side effects such as increased risks of breast and endometrial cancers, sexual dysfunction, cardiovascular risk, and metabolic syndrome (7, 8). Therefore, it is imperative to conduct more mechanism research studies on the protective effect of estrogen in order to improve the efficacy of ERT.

Genome-wide association studies have recently identified many high-risk loci or susceptibility genes for SCZ, including the *MIR137HG* gene (encoding microRNA-137, miR-137) (9, 10). Mounting correlation evidence implicates that miR-137 plausibly exerts an important protective effect on SCZ. MiR-137 is one of the neural tissue-specific miRNAs (11). Many of its target genes are independently associated with SCZ (12–14). MiR-137 can control neural synaptic function by regulating synaptogenesis, maturation, and conduction (15, 16). The haplotype or genotype with the low efficacy of miR-137 expression is the risk factor for SCZ (17, 18). However, based on the single nucleotide polymorphism (SNP) rs1625579, the SCZ-protective effect of miR-137 cannot be consistently confirmed in case–control studies. Even in the same Han population, there are both positive (17, 18) and negative (19, 20) reports. Fortunately, our previous case–control study based on other two functional SNPs rs1198588 and rs2660304 has confirmed the plausible protective effect of miR137 (21). However, the SCZ-protective effect of miR-137 may be female specific. However, our observation is relatively believable since the negative symptom scores and the total Positive and Negative Symptom Scale (PANSS) scores are significantly higher only in female patients carrying the risk genotype of SNP rs1625579 (22). Therefore, it can be speculated that female sex hormones are involved in the SCZ-protective effect of miR-137. Recently, the interactions between heredity and environmental factors in SCZ have received much attention (23, 24). In fact, most of these SCZ-associated high-risk loci are common mutation loci, and the association efficiency of each change point is small (9), which means that each genetic change to take effect needs cooperation with many other genetic risk factors or enhancement by environmental factors. Coincidently, female sex hormones and miR-137 that we focused on here are environmental and genetic factors, respectively.

In this study, based on the well-known SNP rs1625579, a plausible female-specific protective effect of miR-137 was also marginally found. Estrogen (17β-estradiol, E2) upregulated the expression of miR-137 *in vitro*. Both serum E2 and blood miR-137 were significantly decreased or downregulated in SCZ patients. The upregulation of miR-137 by E2 might be abrogated by elevated prolactin (PRL) in female patients. These results suggest that the SCZ-plausible protective effect of miR-137 potentially involves estrogen, and further suggest some new genetic/environmental interaction-related pathophysiology of female SCZ, implying some promising ways to treat female SCZ or to promote the efficacy of ERT in future.

## Materials and methods

### Subjects

A total of 1,004 SCZ patients and 896 healthy controls were enrolled, and their peripheral anticoagulant blood was routinely collected to extract genomic DNA for SNP genotyping as mentioned in a previous report (21). The demographic characteristics are shown in Supplementary Table 1. SCZ was diagnosed according to the Diagnostic and Statistical Manual of Mental Disorders (DSM-V), and patients' clinical manifestations and cognitive function were assessed using the PANSS and the Brief Assessment of Cognition in Schizophrenia (BACS), respectively. In order to study the relationships between female sex hormones and miR-137, 41 SCZ treatment-naïve patients and 43 healthy controls were consecutively enrolled from the Affiliated Hospital of Guangdong Medical University from April 2020 to September 2021. Patients or healthy controls with severe physical illness, pregnancy, or usage of sex hormones or contraceptive drugs were excluded. None of the healthy controls had a personal or family history of major mental disease or substance abuse. Their demographic characteristics are shown in Supplementary Table 2. Peripheral anticoagulant and non-anticoagulant blood as well as basic clinical data were collected. Peripheral anticoagulant blood was used for miRNA quantifications, and non-anticoagulant blood was sent to a clinical lab for the detection of serum E2 and PRL.

### SNP genotyping of miR-137 gene

SNP rs1625579 (T/G) of the miR-137 gene were genotyped by the improved multiple link detection response (iMLDR, Genesky Biotechnologies Inc., Shanghai, China) assay as described previously (21). Genotypes and alleles were analyzed using GeneMapper 4.1 software. Haplotypes were constructed using Haploview 4.2 software. Only those haplotypes with frequencies >3% were further analyzed.

### Cell lines and E2 treatment

The sensitivity of human tissues to E2 varies greatly. The breast and endometrium are sex hormone-sensitive tissues. Here, the human mammary adenocarcinoma cell line (MCF-7) was used to represent traditional sex hormone-sensitive tissues. On the other hand, SCZ is a central nervous disease. Therefore, the mouse hippocampal neuron cell line (HT22) was used to represent human neural tissues. These cell lines were purchased from the Cell Resource Center, Shanghai Institute of Biological Sciences, and Chinese Academy of Sciences and were routinely cultivated in high-glucose DMEM medium with 10% fetal bovine serum (Thermo Fisher Scientific Co., LTD, Shanghai, China). E2 (Sigma-Aldrich, Shanghai, China) was used as an estrogen representative. The cytotoxicity test (CCK8, Dojindo Laboratories, Japan) was performed on these cell lines after treatment at 0–10 nM E2 for 6 h, a peak time of the effect of E2 on miRNAs (25). The similarly treated cells were finally collected for miRNA quantifications.

### MiRNA quantifications

Total RNAs of E2-treated cells and the peripheral anticoagulant blood samples were extracted using the HiPure Blood RNA Mini kit (Magen Biotechnology Co., Ltd, Guangzhou, China). MiRNAs in these samples were detected by the real-time fluorescent quantitative reverse transcription-PCR kit (Exodiagnosis Biotechnology Co., Ltd, Guangzhou, China). The reverse transcription of miRNAs was conducted using a stem-loop primer method. The miRNAs and their corresponding primers for reverse transcription and PCR amplification are shown in Supplementary Table 3. In order to quantify miR-137 accurately, we used three reference miRNAs that have been reported to be elevated (miR-195), decreased (miR-34a), or uncertainly regulated (miR-181b) after E2 treatment *in vitro* (26–28). Each test *in vitro* had three parallel wells and was repeated twice. Each peripheral blood sample was detected three times, and the mean was used for statistical analyses.

### Statistical analysis

The continuous variables were presented as the mean ± standard deviation (SD) and compared by student's *t*-test between the two independent groups. Pearson's chi-square test was used to assess the Hardy–Weinberg equilibrium and the differences in genotypic and allelic distributions between patients and controls. Generalized odds ratios (ORs) with 95% confidence intervals (CIs) of the alleles were also calculated. The correlations between miR-137 and E2 or PRL were analyzed by Spearman correlation analysis. A *P*-value of < 0.05 was considered to be statistically significant. Statistical analysis was performed using SPSS software (version 21.0).

## Results

### SCZ-protective effect of miR-137 based on SNP rs1625579

Our previous case–control study has found a female-specific protective effect of miR-137 based on two functional SNPs rs1198588 and rs2660304 (21). However, such female-related characteristics of miR-137 were not found by many other scholars based on SNP rs1625579 (17–20). In order to ascertain the female-specific protective effect of miR-137, furthermore, SNP rs1625579 was genotyped in a large sample size of the Han population. The distributions of genotypes and alleles were not significantly different between total SCZ patients and healthy controls, but the distributions of protective GG/GT genotypes and G allele were marginally significantly lower in female patients than those in female healthy controls (Table 1), which existed regardless of the difference in gender distribution (Supplementary Table 1) since similar *P*-value was obtained after adjustment of the sex percentage of the patient group according to the control group (Supplementary Table 4). Therefore, although the genetic association with SCZ varies greatly as given SNPs, the female-related characteristic of the protective effect of miR-137 truly exists based on those genetic association studies.

**Table 1:** Genotype and allele frequencies of SNP rs1625579 in miR-137 gene in schizophrenic patients and healthy controls.

**rs1625579**	***n***	**Genotype**, ***n*** **(%)**		**χ2**	***P***	**Allele**, ***n*** **(%)**		**χ2**	***P***	**OR**	**95%CI**	**χ2-HWE**	***P-HWE***
		**TT**	**TG + GG**			**T**	**G**						
**Total**													
Patients	1,004	898 (89.4)	106 (10.6)	2.17	0.141	1,898 (94.5)	110 (5.48)	2.47	0.116	1.24	0.95–1.62	0.36	0.547
Control	896	782 (87.3)	114 (12.7)			1,672 (93.3)	120 (6.70)						
**Male**													
Patients	634	559 (88.2)	75 (11.8)	0.33	0.564	1,190 (93.9)	78 (6.15)	0.46	0.496	1.12	0.81–1.56	0.17	0.670
Control	533	464 (87.1)	69 (12.9)			993 (93.2)	73 (6.85)						
**Female**													
Patients	370	339 (91.6)	31 (8.37)	3.18	0.074	708 (95.7)	32 (4.32)	3.32	0.068	1.53	0.97–2.43	0.15	0.699
Control	363	318 (87.6)	45 (12.4)			679 (93.5)	47 (6.47)						

*n*, number individuals or alleles; OR, odds ratio; CI, confidence interval; HWE, Hardy–Weinberg equilibrium.

### E2 upregulated miR-137 expression *in vitro*

**Figure 1:**
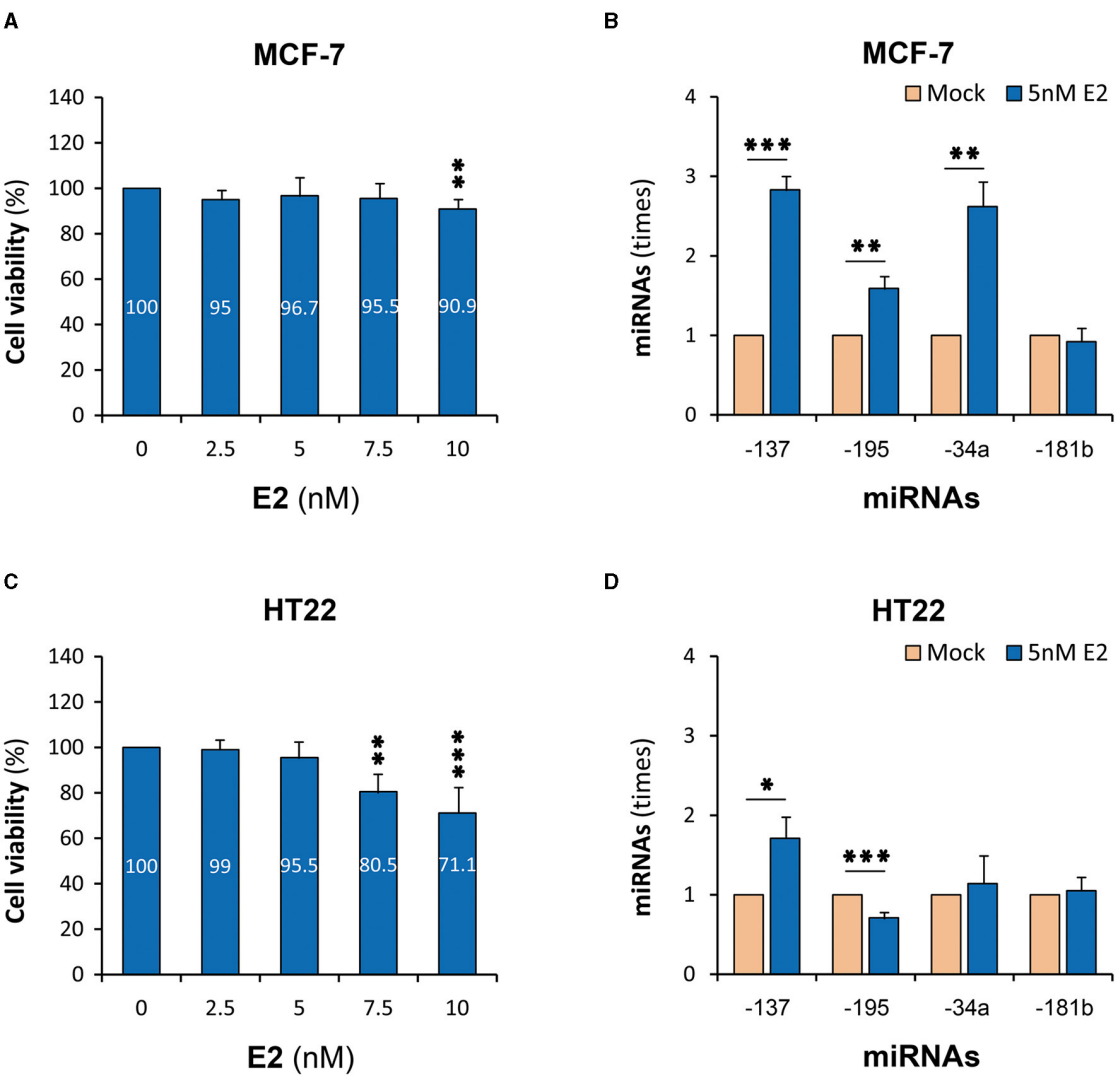
E2-upregulated miR-137 expression in cell lines. MCF-7, human mammary adenocarcinoma cell line; HT22, mouse hippocampal neuron cell line; E2, 17β-estradiol; miRNAs, microRNAs. **(A)** Cell viability test of MCF-7 cells. **(B)** MiR-137 expression in E2-treated MCF-7 cells. **(C)** Cell viability test of HT22 cells. **(D)** MiR-137 expression in E2-treatd HT22 cells. ^*^P < 0.05, ^**^P < 0.01, ^***^P < 0.001.

Both MCF-7 and HT22 cells constitutively expressed detectable levels of miR-137 and other three miRNAs (Supplementary Figure 1), implying that these cells were suitable for the study on the relationship between E2 and miR-137. Cytotoxicity tests showed that the maximum concentration of E2, which let the viability of both cells maintain above 95%, was 5 nM when the treatment time was 6 h (Figures 1A, C). Under such treatment parameters, the expression of miR-137 in MCF-7 cells was upregulated to 2.83 times (*P* < 0.001) (Figure 1B), and that in HT22 cells was upregulated to 1.81 times (*P* < 0.05) (Figure 1D). Among three reference miRNAs, miR-195 and miR-34a were significantly upregulated in MCF-7 cells but were, respectively, downregulated and remained unchanged in HT22 cells. As for miR-181b, it remained unchanged in both MCF-7 and HT22 cells.

### Dysregulations of serum E2 and blood miR-137 in SCZ patients

**Figure 2:**
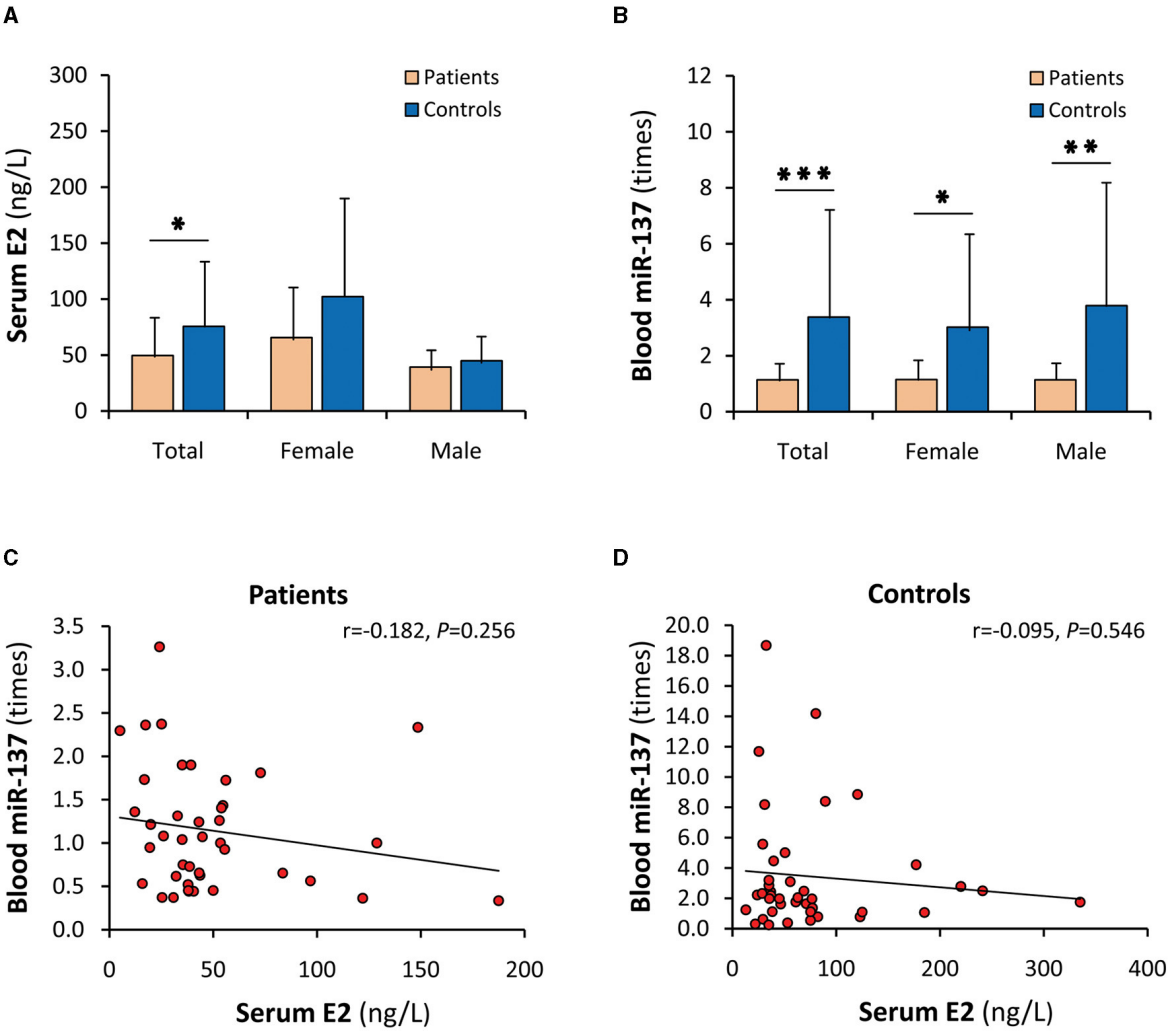
Dysregulations of serum E2 and blood miR-137 in SCZ patients. E2, 17β-estradiol; miR-137, microRNA-137. Serum E2 **(A)** and blood miR-137 **(B)** in SCZ patients and healthy controls. Correlation analyses of serum E2 with blood miR-137 in SCZ patients **(C)** and healthy controls **(D)**. ^*^P < 0.05, ^**^P < 0.01, ^***^P < 0.001.

Both serum E2 and blood miR-137 in patients were significantly decreased or downregulated (*P* < 0.05 and *P* < 0.001, respectively) (Figures 2A, B). Blood miR-137 in SCZ patients was only 33.7% of that in healthy controls. When stratified by sex, blood miR-137 was significantly different, but serum E2 was not, between patients and controls in males and females, respectively. Furthermore, serum E2 was not correlated with blood miR-137 in both SCZ patients and healthy controls (Figures 2C, D).

### Gender influences on the correlation of serum E2 with blood miR-137

**Figure 3:**
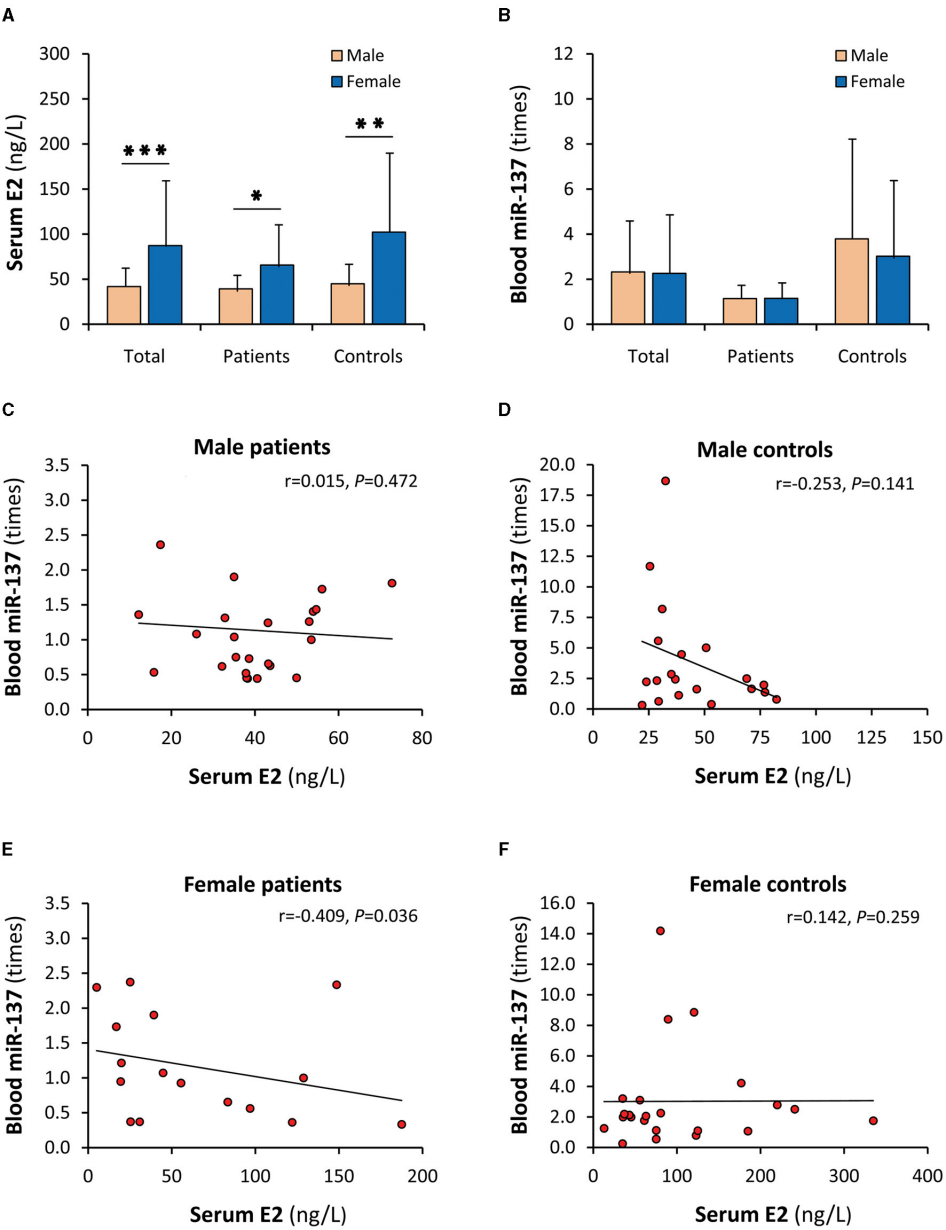
Gender influence on the correlation of serum E2 with blood miR-137. E2, 17β-estradiol; miR-137, microRNA-137. Serum E2 **(A)** and blood miR-137 **(B)** in male and female individuals. Correlation analyses of serum E2 with blood miR-137 in male SCZ patients **(C)**, male healthy controls **(D)**, female SCZ patients **(E)**, and female healthy controls **(F)**. ^*^P < 0.05, ^**^P < 0.01, ^***^P < 0.001.

Although serum E2 and blood miR-137 were synchronously decreased in SCZ patients, their uncorrelations in patients and healthy controls were not concordant with the *in vitro* results that E2 upregulated the expression of miR-137. A possible explanation is that gender significantly affects the relationship between serum E2 and blood miR-137. Indeed, when stratified by sex, serum E2 in females was significantly higher than males in both patients and controls (Figure 3A), but blood miR-137 was not different in either patients or controls (Figure 3B). Obviously, serum E2 did not upregulate miR-137 expression *in vivo*, suggesting that there was an unknown factor that abrogated the miR-137 upregulation effect of E2 in females. Moreover, serum E2 was not correlated with blood miR-137 in male patients and male and female healthy controls (Figures 3C, D, F) but was negatively correlated with blood miR-137 in female patients (Figure 3E), suggesting that the unknown factor is dominant in female patients.

### Dysregulation of serum PRL in SCZ patients

**Figure 4:**
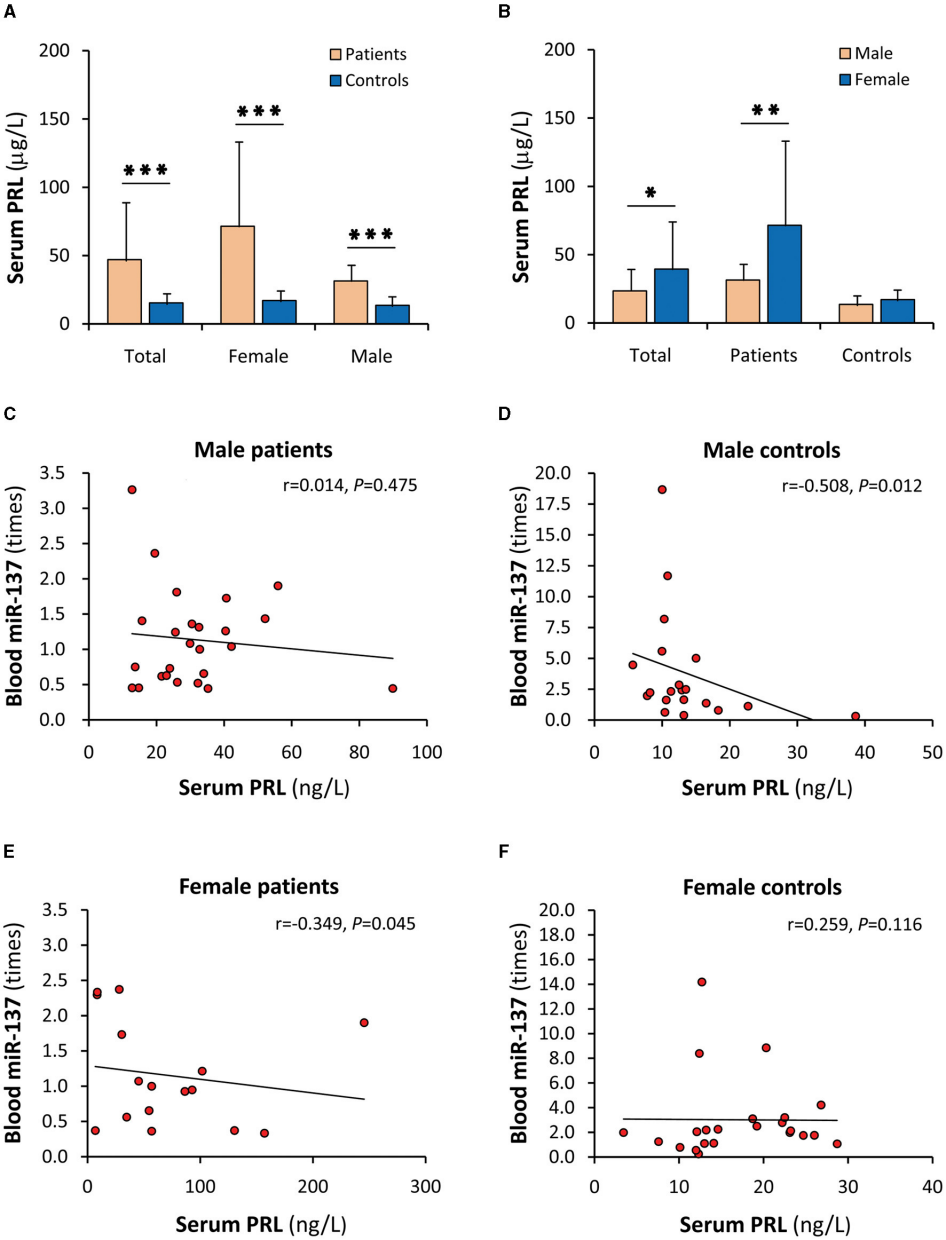
Serum PRL and its correlations with blood miR-137. PRL, prolactin; miR-137, microRNA-137. Serum PRL in SCZ patients and healthy controls **(A)**, and in males and females **(B)**. Correlation analyses of serum PRL with blood miR-137 in male SCZ patients **(C)**, male healthy controls **(D)**, female SCZ patients **(E)**, and female healthy controls **(F)**. ^*^P < 0.05, ^**^P < 0.01, ^***^P < 0.001.

The level and effect of E2 *in vivo* may be affected by PRL (3), and PRL is also an important SCZ-related sex hormone (29, 30). For these reasons, PRL was speculated as the above unknown antagonistic factor and was detected in this study. As a result, PRL was significantly increased in SCZ patients (Figure 4A). There was also a significant difference between patients and controls in both males and females. When stratified by sex, the increase in serum PRL mainly occurred in female patients (Figure 4B). Furthermore, serum PRL was uncorrelated with blood miR-137 in both patients and healthy controls (Supplementary Figures 2A, B). It was also uncorrelated with blood miR-137 in male SCZ patients and female healthy controls (Figures 4C, F) but was negatively correlated with blood miR-137 in male healthy controls (Figure 4D) and female SCZ patients (Figure 4E). The negative correlation with blood miR-137, together with the increased serum level, implies that PRL indeed acted as an antagonist in female patients.

## Discussion

Increasing correlation evidence has shown that miR-137 is deeply involved in the pathophysiology of SCZ (9–15). However, the underlying mechanisms remain elusive. Our previous study has shown that miR-137 plays an SCZ-protective role in a female-specific manner based on two functional SNPs rs1198588 and rs2660304 (21). In this study, a marginal female-specific protective effect of miR-137 was also found based on the well-known SNP rs1625579. Furthermore, E2 was found to upregulate the expression of miR-137 in sex hormone-sensitive tissues representing MCF-7 cells and neural tissues representing HT22 cells, and both serum E2 and blood miR-137 were significantly decreased or downregulated in SCZ patients. On the other hand, the negative correlation of serum E2 with blood miR-137, along with significantly higher PRL in female patients, implies that miR-137 might be upregulated by E2 but was potentially abrogated by PRL in female patients. Therefore, it is suggested that the underlying mechanism of the plausible female-specific SCZ-protective effect of miR-137 involves estrogen, or miR-137 upregulation may be one of the underlying mechanisms for estrogen to protect normal females against SCZ. In female SCZ patients, however, such protective effects of E2/miR-137 may be abrogated possibly by the elevated PRL. These preliminary findings imply some new ways to treat female SCZ or to promote the efficacy of ERT in future.

SNP rs1625579 of the miR-137 gene is one of the strongest genetic variant predictors of SCZ in GWAS (10, 24, 31). Its T allele is identified as the major allele and the risk allele for SCZ, implying that the minor G allele appears to be SCZ-protective. The miR-137 levels are lower in the brains of neurotypical subjects homozygous for the risk T allele (32). In contrast, the protective minor G allele is associated with elevated miR-137 expression *in vitro* (14, 33). Therefore, most authors believe that miR-137 itself is SCZ-protective. However, the genetic correlation of SNP rs1625579 with SCZ is not consistently confirmed in large-scale case–control studies (17–20), and the risk allele is highly prevalent in a healthy population, suggesting that miR-137 dysregulation alone is highly unlikely to cause SCZ, but miR-137 may influence how the brain responds to other genetic and environmental risk factors for SCZ (31). Coincidently, our previous study has shown that miR-137 plays a plausible protective role in a female-specific manner based on genetic association analyses (21). Here, this unique manner was inconclusively confirmed by SNP rs1625579 genotyping though no definite genetic correlation with SCZ was found. Along with that, the negative symptom scores and the total PANSS-score are significantly higher only in females carrying the risk genotype of SNP rs1625579 (22), and these studies strongly suggest that female sex hormones as environmental factors are involved in the underlying mechanism of the plausible SCZ-protective effect of miR-137.

E2 is the major form of estrogen. In this study, E2 was found to increase the expressions of miR-137 in both MCF-7 and HT22 cells, which is consistent with the reports that estrogen can upregulate or inhibit the expressions of other miRNAs (25–27). It is very difficult to directly study miR-137 in the nervous system of SCZ patients, and there is yet a lack of typical animal models of SCZ. Therefore, the mouse hippocampal neuron cell line, HT22 (34), a relatively feasible model for neuron research, was used and confirmed to express miR-137 constitutionally, similar to primary mouse hippocampal neurons (35). Combined with the fact that estrogen alters the expression of many miRNAs in the ventral and dorsal hippocampal gyrus of rats in an age- and region-specific manner (36), it is suggested that the miR-137 upregulation by E2 may take an important part in the pathophysiology of SCZ. In other words, it is one of the underlying mechanisms for estrogen to protect normal females against SCZ. On the other hand, E2 did not drastically upregulate the expression of miR-137 *in vitro*, which is in concordance with the small association efficiency of each genetic change and the deficiency for miR-137 to cause SCZ alone (9, 31). However, the moderate miR-137 upregulation by E2 reasonably explains the female-specific protective effect of miR-137 in normal females since the subtle expression superiority of the protective genotype or allele may be continuously enhanced by plenty of estrogens from embryogenesis to before menopause in females. This new genetic/environmental interaction mechanism also explains why the dysregulation of miR-137 is not usually detected in the postmortem brains of SCZ patients (31).

In order to clarify the significance of the miR-137 upregulation by E2 in SCZ patients, serum E2 and peripheral blood miR-137 in SCZ patients and healthy controls were studied. Compared with healthy controls, both serum E2 and blood miR-137 were significantly decreased or downregulated in patients. The decrease in E2 in SCZ patients is consistent with the conclusion in the literature (3, 4). However, the downregulation of blood miR-137 is not consistent with the reports by other scholars. There were only four studies (two original studies, one review, and one meta-analysis) related to the expression of miR-137 in the peripheral blood of SCZ patients (37–40). Their conclusions all show a significant upregulation. A possible explanation for this inconsistency is that those studies use plasma rather than whole peripheral blood as samples. Indeed, miRNAs in plasma are different from those in PBMCs (41), and as many as 83 miRNAs in the peripheral blood of patients with SCZ are decreased when detected using chip technology (42). Therefore, the downregulated blood miR-137 found in SCZ patients in this study is believable, and whole peripheral blood miR-137 seems to better reflect the essence of miR-137 deficiency in the brain of SCZ patients. The decrease in both serum E2 and blood miR-137 was consistent with the miR-137 upregulation by E2 in the cell models and also consistent with their same SCZ-protective effects, supporting that the miR-137 upregulation by E2 may play important roles in protecting normal females against SCZ.

Although it well-explains the female-specific protective effect in the individuals carrying the protective minor allele, the miR-137 upregulation by E2 cannot directly explain why most individuals carrying the risk allele do not get sick. For this reason, the relationship between serum E2 and blood miR-137 in SCZ patients and healthy controls were further investigated. However, between serum E2 and blood miR-137, uncorrelations rather than the expected positive correlations were found in both patients and healthy controls. When stratified by sex, the high levels of serum E2 did not lead to elevated blood miR-137 expression in female patients and female healthy controls and even serum E2 was negatively correlated with blood miR-137 in female patients, suggesting that miR-137 upregulation by E2 is abrogated by some antagonistic factors *in vivo*. The assumptive antagonist may be PRL since the level and effect of E2 may be affected by PRL *in vivo* (3). Indeed, serum PRL was significantly increased in SCZ patients as reported (29). When stratified by sex, female patients had the highest serum PRL level and a negative correlation between serum PRL and blood miR-137, suggesting that some females are still subjected to SCZ since the elevated PRL potentially abrogates the plausible protective effect of the miR-137 upregulation by E2. Therefore, the PRL blockage of the miR-137 upregulation by E2 may form a novel E2/PRL/miR-137-related pathophysiology of female SCZ. On the other hand, it is not known why serum PRL in males, unlike in females, was negatively correlated with blood miR-137 in healthy controls rather than in SCZ patients. The possible explanation is that males develop into SCZ in different manners. Consistently, SCZ-biased genes diverge between males and females when analyzed by single-cell sequencing data (43).

The present study has several limitations. First, the cell lines MCF-7 and HT22 are cancer cells and animal cells, respectively. They are not ideal models of brain physiology. Further studies are needed to be carried out in more representative human cell lines such as induced or transdifferentiated pluripotent stem cells (44, 45). Second, the human sample used for correlation analyses between miR-137 levels and sex hormones in this study was small and was limited to the Han Chinese population. Thus, our findings and conclusions in this study were preliminary. Since our patients' results originated only from correlations, other approaches that can identify causality should be used in future. In addition, further mechanism studies on animal models and in a large sample of human subjects are needed to clarify the influences of sex hormones on miR-137 expression and the significance of such genetic/environmental interaction mechanisms in the pathophysiology of SCZ.

## Conclusion

The plausible female-specific SCZ-protective effect of miR-137 potentially depends on the female sex hormone estrogen, of which the underlying mechanism may be that E2 upregulates the expression of miR-137. However, some females are still susceptible to SCZ since the plausible protective effect of the miR-137 upregulation by E2 may be abrogated by the elevated PRL. Although more causality identification and mechanism studies are imperative in future, our findings suggest a new genetic/environmental interaction mechanism for E2/miR-137 to protect normal females against SCZ and a novel E2/PRL/miR-137-related pathogenesis of female SCZ and imply some new ways such as ERT combined with PRL-lowering drugs to treat female SCZ in future.

## Data availability statement

The original contributions presented in the study are included in the article/Supplementary material, further inquiries can be directed to the corresponding authors.

## Ethics statement

The studies involving humans were approved by Ethics Committee of Affiliated Hospital of Guangdong Medical University. The studies were conducted in accordance with the local legislation and institutional requirements. The participants provided their written informed consent to participate in this study.

## Author contributions

All authors listed have made a substantial, direct, and intellectual contribution to the work and approved it for publication.
