# Supplementary material for: Schizophrenia plausible protective effect of microRNA-137 is potentially related to estrogen and prolactin in female patients
Source: Front Psychiatry. 2023 Aug 23;14:1187111. doi: 10.3389/fpsyt.2023.1187111 (PMC10482089; doi:10.3389/fpsyt.2023.1187111)
Supplement: Supplementary file 1 [file Data_Sheet_1.docx]

Supplementary Material

Schizophrenia-protective effect of microRNA-137 involves estrogen and is abrogated possibly by elevated prolactin in female patients

Qian Peng ^1†^, Zhun Dai ^1†^, Jingwen Yin ^1^, Dong Lv ^1^, Xudong Luo ^1^, Susu Xiong, Zhijiang Yang ^1^, Guangmin Chen ^1^, Yaxue Wei ^1^, Ying Wang ^1^, Dandan Zhang ^1^, Lulu Wang ^1^, Debo Yu ^1^, Yusheng Zhao ^1^, Dele Lin ^1^, Zhiyu Liao ^1^, Yongxi Zhong ^1^, Zhixiong Lin ^1*^, Juda Lin ^1*^

*** Correspondence:** Juda Lin: linjuda@tom.com; Zhixiong Lin: zhixionglinzj@163.com

# Supplementary Figures and Tables

## Supplementary Tables

**Supplementary Table 1.** The demographic characteristics of samples for miR-137 genotyping

| **Variables** | **Patients**  **(n=1004)** | **Controls**  **(n=896)** | **P** |
| --- | --- | --- | --- |
|  |  |  |  |
| Age, mean±SD (years) | 34.8±13.7 | 34.9±9.78 | 0.868 |
| Gender, n (%) |  |  | 0.109 |
| Male | 634 (63.1) | 533 (59.5) |  |
| Female | 370 (36.9) | 363 (40.5) |  |

n, number; SD, standard deviation.

**Supplementary Table 2.** The demographic characteristics of samples for studies on sex hormones and miRNAs expression

| **Variables** | **Patients**  **（n=41）** | **Controls**  **（n=43）** | ***P*** |
| --- | --- | --- | --- |
| Age, mean±SD (years) | 30.5±11.6 | 31.3±10.6 | 0.762 |
| Gender, n (%) |  |  | 0.183 |
| Male | 25 (61.0) | 20 (46.5) |  |
| Female | 16 (39.0) | 23 (53.5) |  |

n, number; SD, standard deviation.

**Supplementary Table 3.** miRNAs species and reverse transcription and PCR primers

| **miRNAs** | **Function** | **Sequence (5'→3'）** | **Size (bp)** |
| --- | --- | --- | --- |
| hsa-miR-137-3p | F | ACACTCCAGCTGGGTTATTGCTTAAGAATACG | 72 |
|  | RT | CTCAACTGGTGTCGTGGAGTCGGCAATTCAGTTGAGCTACGCGT |  |
| hsa-miR-195-5p | F | ACACTCCAGCTGGGTAGCAGCACAGAAATATT | 75 |
|  | RT | CTCAACTGGTGTCGTGGAGTCGGCAATTCAGTTGAGGCCAATAT |  |
| hsa-miR-34a-5p | F | ACACTCCAGCTGGGTGGCAGTGTCTTAGCTGGT | 71 |
|  | RT | CTCAACTGGTGTCGTGGAGTCGGCAATTCAGTTGAGACAACCAG |  |
| hsa-miR-181b-5p | F | ACACTCCAGCTGGGAACATTCATTGCTGTCGG | 73 |
|  | RT | CTCAACTGGTGTCGTGGAGTCGGCAATTCAGTTGAGACCCACCG |  |
| mmu-miR-181b-5p | F | ACACTCCAGCTGGGAACATTCATTGCTGTCGGT | 74 |
|  | RT | CTCAACTGGTGTCGTGGAGTCGGCAATTCAGTTGAGAACCCACCG |  |
| U6 | F | CTCGCTTCGGCAGCACA | 94 |
|  | R | AACGCTTCACGAATTTGCGT |  |
|  | RT | AACGCTTCACGAATTTGCGT |  |

has, human; mmu, mouse; F, forward; RT, reverse transcription; R, reverse.

**Supplementary Table 4.** Pearson's chi-square test of genotype and allele frequencies of SNP rs1625579 with adjustment of sex distribution

| **Variables** | ***P*^C^** |
| --- | --- |
| Genotype |  |
| Total | 0.140 |
| Female | 0.079 |
| Allele |  |
| Total | 0.112 |
| Female | 0.069 |

*P*^C^, *P* values after adjustment of the sex percentage of the patient group according to the control group.

## Supplementary Figures

**Supplementary Figure 1.** Constitutive expression of miR-137 in MCF-7 and HT22 cells. MCF-7, human mammary adenocarcinoma cell line; HT22, mouse hippocampal neuron cell line; E2, 17β-estradiol.*P<0.05, **P<0.01, ***P<0.001, HT22 vs MCF-7 cells.


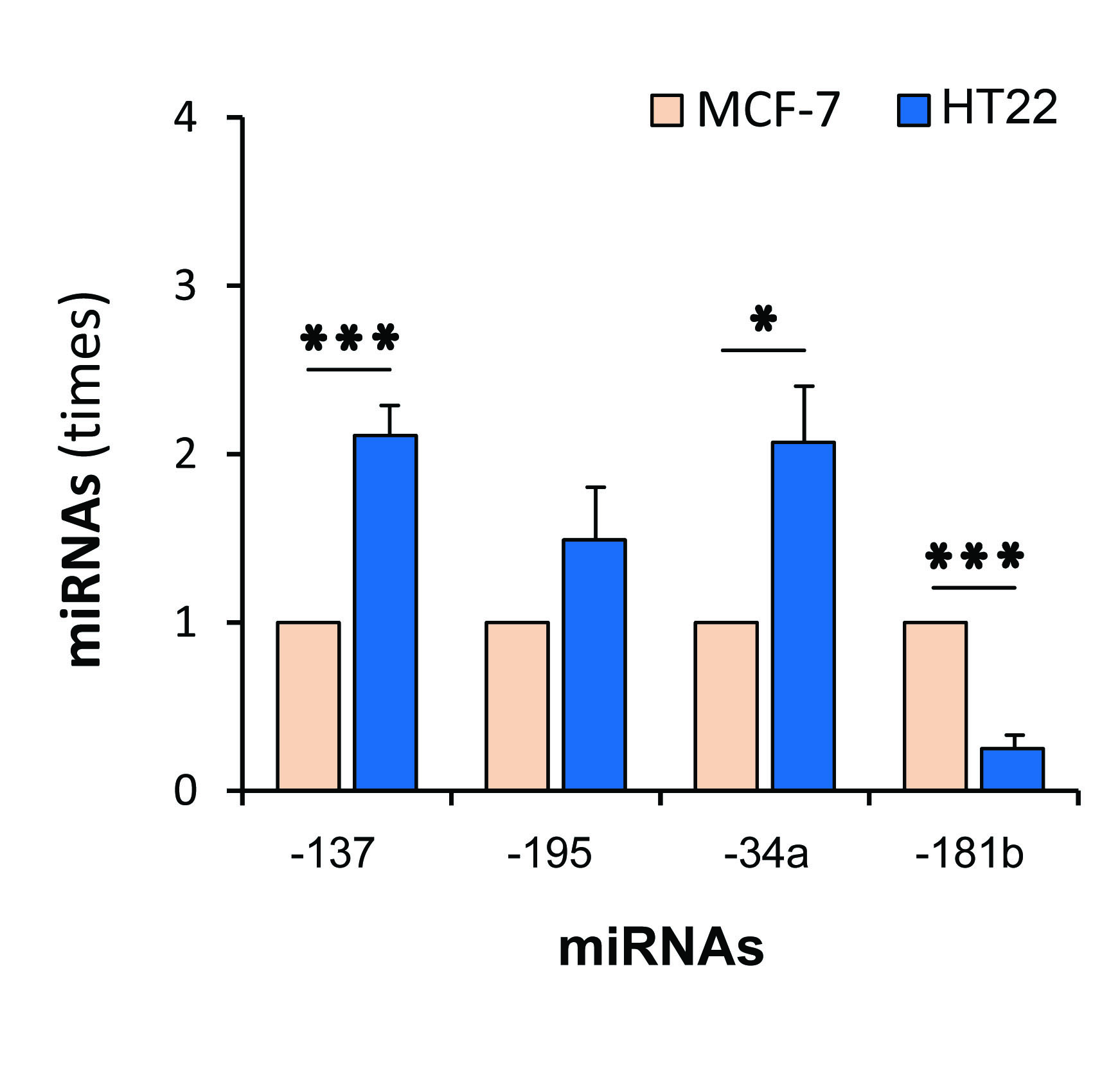

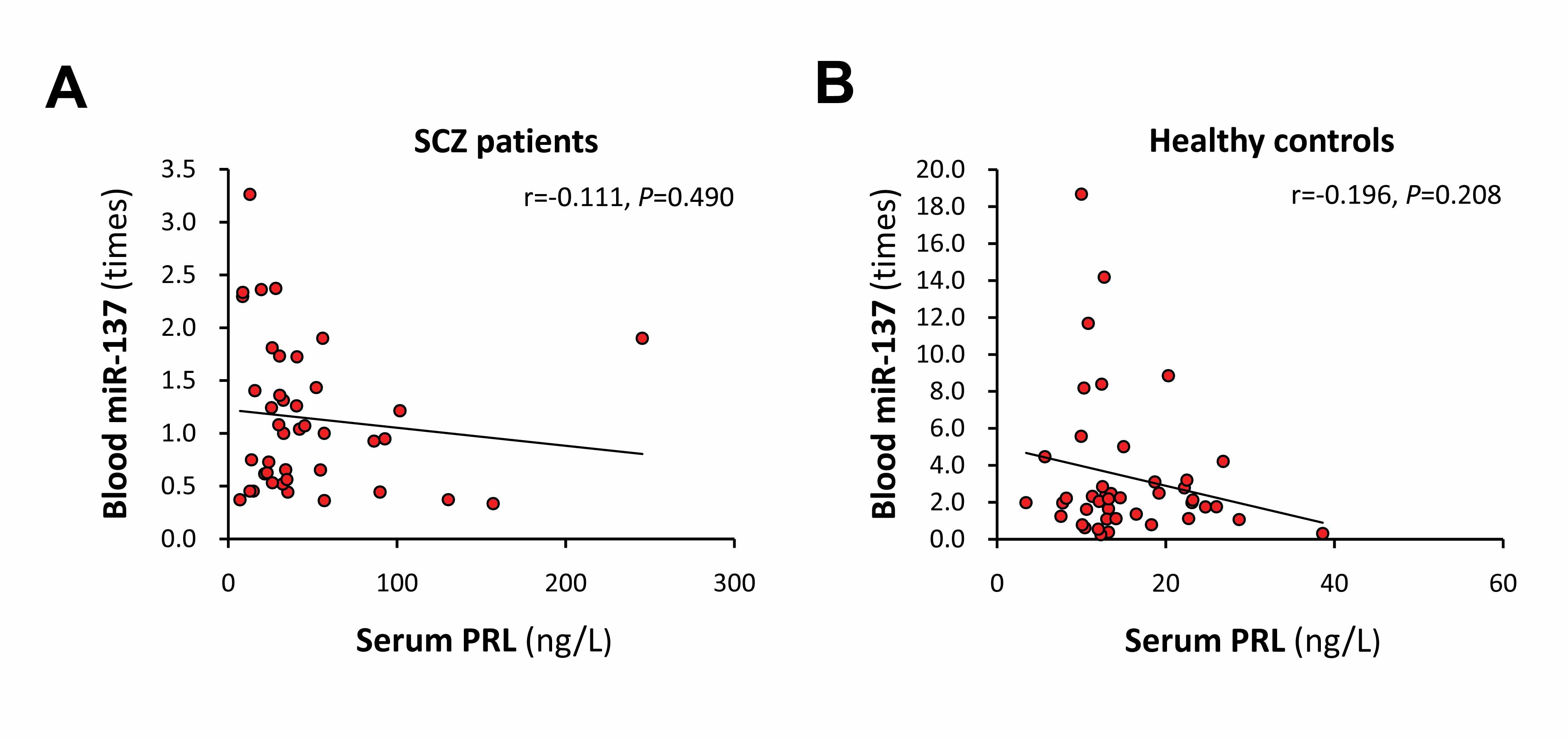


**Supplementary Figure 2.** Correlation of serum PRL with blood miR-137. SCZ, Schizophrenia; PRL, prolactin; miR-137, microRNA-137. Correlation analyses of serum PRL with blood miR-137 in SCZ patients (A) and healthy controls (B).
